# Guiding medical trainees' workplace learning for interprofessional collaboration—Looking to physicians or seeing nurses?

**DOI:** 10.1111/medu.15617

**Published:** 2025-02-08

**Authors:** Renée E. Stalmeijer, Willem S. de Grave, Frank W. J. M. Smeenk, Lara Varpio

**Affiliations:** ^1^ School of Health Professions Education, Department of Educational Development and Research, Faculty of Health, Medicine and Life Sciences Maastricht University Maastricht the Netherlands; ^2^ Catharina Hospital Eindhoven the Netherlands; ^3^ Department of Pediatrics Perelman School of Medicine at the University of Pennsylvania Philadelphia Pennsylvania USA; ^4^ Children's Hospital of Philadelphia Philadelphia Pennsylvania USA

## Abstract

**Introduction:**

Effective healthcare practice requires interprofessional collaboration (IPC) between all members of the healthcare team. Preparing healthcare trainees for IPC has proven to be difficult. Research suggests that workplace learning has a vital role in acquiring IPC competencies. However, guidance is required for trainees to make optimal use of IPC workplace learning opportunities. This study asks (1) To what extent is guidance provided to medical trainees when learning to collaborate interprofessionally?; and (2) Who provides this guidance and how?

**Methods:**

We conducted a constructivist, qualitative study using iterative cycles of semi‐structured interviews and reflexive thematic analysis. A purposive sample of medical trainees (N = 11), nurses (N = 9), advance practice clinicians (APCs)(N = 3) and attending physicians (N = 9) associated with a department of general internal medicine in a non‐academic teaching hospital participated in this study. Data‐analysis was informed by Billett's concept of guidance.

**Results:**

Guidance on IPC was provided to medical trainees both *intra*professionally and *inter*professionaly, be it limited, informal and implicit. Nurses and APCs provided more guidance on IPC than attending physicians. Guidance by attending physicians included implicit modelling and only became explicit in response to concerns raised about trainees' IPC by nurses. Nurses and APCs provided continuous guidance on various aspects of IPC but felt hampered by hierarchical issues to provide feedback. Trainees were more focused on feedback provided by attending physicians than they were aware of nurses' feedback.

**Conclusion:**

The potential of workplace learning to learn IPC competencies is currently hampered by a lack of explicit guidance. Optimizing the role of workplace learning in IPC competence development requires more explicit role modelling and collaborative intentionality by attending physicians, more critical reflection by trainees and a strengthening of the feedback role of nurses and APCs.

## Abstract 2

#Workplacelearning is vital in acquiring interprofessional competences. However, guidance is required for trainees to optimally use IPC learning opportunities, begging the questions of does guidance on IPC occur, how and by whom?

## 1 INTRODUCTION

Interprofessional collaboration (IPC) is celebrated for enabling high‐quality patient care.^1^, ^2^ In response, health professions education (HPE) curricula often champion IPC as an essential competence for healthcare trainees.^2^, ^3^, ^4^ To date, Interprofessional Education (IPE) has been the preferred educational approach for fostering collaboration‐ready professionals.^3^ However, research suggests that IPE has yet to meet the challenge^5^; IPE has been dismissed as a tick‐box activity implemented merely to please accreditation committees.^6^ Recent work reports that trainees still feel ill‐equipped for IPC.^7^

In 2018, Paradis and Whitehead argued that the timing of IPE implementation in HPE curricula may limit the effectiveness and transfer of IPC skills to clinical practice.^5^ IPE is often operationalized through undergraduate educational activities during which learners from different health professions learn with, from and about each other.^2^ Such early implementation is reasoned to help circumvent the establishment and consolidation of professional stereotypes, thus encouraging future clinicians to be more receptive to and respectful of the skills and judgements of their interprofessional colleagues.^8^, ^9^, ^10^ However, several scholars question this reasoning.^5^ Michalec and Hafferty^8^ suggest that students must first develop a clear understanding of their own professional identity and scope of practice before being expected to make sense of the scope of practice of other healthcare team members. Accordingly, some researchers suggest that learning to collaborate interprofessionally might be more effective during workplace learning.^5^, ^11^, ^12^

Workplace learning is vitally important to the education of medical professionals.^13^ Throughout the medical education continuum, both undergraduate and postgraduate medical trainees undergo various clinical rotations during which they learn to engage in clinical practice and to participate in the daily practices of healthcare.^14^ From a socio‐cultural lens, workplaces powerfully shape medical trainees' understanding of their roles and responsibilities within healthcare teams, and immerse them in the healthcare systems they will need to navigate during practice.^15^ During workplace learning, medical trainees ideally learn what it means to be a member of both the community of physician practice and the landscape of healthcare practice.^12^, ^16^ By experiencing authentic clinical practice, trainees develop knowledgeability about how the healthcare system and professional hierarchy shape practice and professional relationships.^5^, ^15^ However, research suggests that learning in the clinical workplace is driven by the demands of healthcare (including, for example, patient safety concerns, patient turnover, staff and medical resources) and not the needs of trainee learning,^17^ Thus, it is not surprising that a large body of research points to the necessity of supervision,^18^ intentional teaching^19^ and guidance^13^ during workplace learning.

While the *formal* supervision and teaching of medical trainees engaging in workplace learning is bound by regulations and is explicitly assigned to senior physicians, the less formal *guiding* of trainees could be carried out by any experienced member of the workplace environment.^20^ When it comes to learning to collaborate interprofessionally, scholars have argued that non‐physician members of the healthcare team like nurses,^21^, ^22^ advanced practice clinicians (APCs)^23^ and allied healthcare professionals^24^ have a role at least equally important in guiding as physicians.^12^ Described in Billett's *Workplace Pedagogy*, guiding is defined as a process through which more experienced members of a workplace guide novice employees' development towards being effective members of a workplace.^20^ Billett argued that this guidance could take many forms including: enabling active participation in workplace practices; identifying and creating access to learning opportunities; modelling skills and behaviours; and socializing trainees into the idiosyncratic work processes and agreements of a setting.^13^, ^20^, ^22^, ^25^ Although *supervision* and *clinical teaching*’ are terms more commonly used in HPE to describe the role of educators in the health professions education literature, we have previously argued and continue to assert that the concept of guidance may provide a better lens to capture activities performed by and behaviours of non‐physician members of the healthcare team while they interact with medical trainees to help address competence gaps.^12^ The lens of guidance (as defined by Billett) highlights how other members of the healthcare team are, in relation to medical trainees, more experienced members of the workplace and may have valuable competences that can aid the trainee in becoming an effective member of the landscape of healthcare practice.^12^

Previous research has used the concept of guidance to look at the various types of guiding behaviour that nurses^22^ and advanced practice clinicians^23^ use to guide trainees. However, these studies have focused on guidance of all and any competencies that medical trainees might need. To date, no studies have focused on interprofessional collaborative competencies and knowledgeability specifically. Furthermore, existing studies provide limited insights into the motivations of different professionals that underpin their willingness to provide this guidance to medical trainees. As a result, there is limited understanding of how those most commonly interacting during workplace learning of medical trainees (i.e. medical trainees, attending physicians, nurses and APCs) acknowledge and feel the need to engage in IPC‐focused guidance of medical trainees.

This study set out to explore the perceptions of medical trainees, attending physicians, nurses and APCs, asking: (1) To what extent is guidance provided to medical trainees when learning to collaborate interprofessionally? and (2) Who provides this guidance and how?

## 2 METHODS

### 2.1 Paradigmatic orientation

We conducted a constructivist, qualitative study using iterative cycles of semi‐structured interviews and reflexive thematic analysis to explore participants' experiences and perceptions while being informed by existing literature on IPE, workplace learning and guidance.

### 2.2 Context

The investigation was carried out within the Dutch medical education training continuum, specifically within the Internal Medicine context. Here, undergraduate medical education lasts 6 years of which three are typically spent in clinical practice clerkships. Then, medical trainees generally participate in up to two years of clinical practice to gain work experience (i.e. working as doctors but not engaging in any formal training). Thereafter, the learners move on to postgraduate medical education in Internal Medicine, a curriculum that lasts, on average, 6 years and is rotation‐based. The first four years consist of General Internal Medicine training. The final two years trainees specialize in a specific area of internal medicine (e.g. nephrology or haematology). The majority of medical education in the Netherlands involves competency‐based education programmes and programmatic assessment, and is situated in both academic and non‐academic hospitals. Formal supervision is performed by more senior physician members of the healthcare team.

In response to national and international calls to improve IPC, several initiatives were implemented within our study context to stimulate IPC, i.e. value‐based healthcare, crew resource management and attention to nurse leadership development roles and speaking up.

### 2.3 Participants

Informed by a previous pilot study^26^ and by prior research,^21^ we purposively sampled medical trainees, attending physicians, nurses and advanced practice clinicians (APCs) (i.e. Nurse Practitioners and Physician Assistants) working in an Internal Medicine department in a non‐academic teaching hospital. With the help of FWJMS key informants were identified that suggested potential participants to RES. RES, who did not have a working relationship with any of the participants, contacted potential participants via e‐mail and scheduled interviews via e‐mail and phone. The information letter and the informed consent form were included in the e‐mail. All participants received a €25,‐ gift certificate for an online store to thank them for their time and participation.

To address our research questions and capture our participants' perceptions on the extent to which guidance was provided on IPC throughout medical trainees' workplace learning trajectories and who provided this guidance, we sampled all our participants across various levels of seniority. This decision was informed by a pilot study^26^ which suggested that depending on seniority: medical trainees are variably aware of guidance on IPC; attending physicians are variable prone to guidance on IPC; and nurses and APCs are variably confident to provide guidance on IPC. The final sample consisted of 32 participants: 11 medical trainees (4 junior, 4 intermediate, 3 senior), 9 attending physicians (3 junior, 2 intermediate, 4 senior), 9 nurses (4 junior, 2 intermediate, 3 senior) and 3 APCs (1 intermediate, 2 senior) (see Table 1 for participant demographics).

**TABLE 1:** Participant characteristics.

Participant group	Experience level	Gender (M/F)	Training in IPC (Y/N)	Training in Clin. Teaching (Y/N)
***Trainees***				
Junior: final year medical student engaged in their senior clerkship Intermediate: medical trainees 1–2 years post medical school, engaged in work experience and have not yet entered residency training & residents in their first year of residency training Senior: 2, 3 and 4th year residents completing General Internal Medicine training, not yet entered specialty training (e.g. nephrology, haematology)				
T 1	Senior	F	N	N
T 2	Senior	M	N	N
T 3	Junior	F	N	N
T 4	Junior	M	N	N
T 5	Junior	F	N	N
T 6	Intermediate	F	Y	Y
T 7	Junior	F	N	N
T 8	Senior	M	N	N
T 9	Intermediate	F	N	N
T 10	Intermediate	F	N	Y
T 11	Intermediate	F	N	Y
***Attending Physicians***				
Junior: <10 years' experience Intermediate: 10–15 years of experience Senior: +15 years of experience				
A 1	Senior	M	Y	Y
A 2	Senior	F	Y	Y
A 3	Senior	F	Y	Y
A 4	Junior	F	Y	Y
A 5	Intermediate	F	N	Y
A 6	Intermediate	F	Y	Y
A 7	Junior	F	N	Y
A 8	Junior	M	N	Y
A 9	Senior	M	N	Y
***Nurses***				
Junior: 1–5 years of experience Intermediate: 6–15 years of experience Senior: +15 years of experience				
N 1	Junior	M	N	N
N 2	Intermediate	F	N	N
N 3	Junior	F	Y	Y
N 4	Junior	F	N	N
N 5	Junior	F	N	Y
N 6	Senior	F	N	N
N 7	Senior	F	N	N
N 8	Intermediate	F	N	Y
N 9	Senior	F	N	Y
***APCs*** (Nurse Practitioner [NP] & Physician Assistant [PA])				
Intermediate: 6–15 years of experience Senior: +15 years of experience				
NP 1	Senior	F	N	N
NP 2	Senior	M	N	N
PA 1	Intermediate	M	N	N

### 2.4 Semi‐structured interviews

The interview guides explored how medical trainees learned to collaborate within interprofessional healthcare teams and aimed to identify which individuals within those teams were involved in guiding trainees during this process. Questions probed topics such as the clinical processes in which interprofessional collaboration plays an important role; the expectations of medical trainees' interprofessional collaboration competence; the people involved in guiding medical trainees' collaboration skills; and the formal or informal nature of that process (See Appendix 1 for an example interview guide). A trained and experienced qualitative scholar (WSdG) carried out the interviews in the first language of the participants (i.e. Dutch) between February and August of 2023. At the start of each interview, both verbal and written consent was solicited from participants. Interviews lasted 26–54 min each (with an average length of 42 min). Each interview was audio recorded and transcribed verbatim by a professional transcription agency. All transcripts were given a code to identify the type of participant and their level of seniority (see Table 1). Identifying information (e.g. names of individuals and institutions) were removed by RES after transcription and before analysis.

### 2.5 Data‐analysis

Data analysis happened concurrently with data collection in iterative cycles using reflexive thematic analysis.^27^ Reflexive thematic analysis highlights the active role of the researcher in the process of data‐analysis and allows for the use of sensitizing concepts and frameworks to guide data‐analysis.^27^

We used two subjectivist inductive approaches to shape this study. First, using a fully theory‐informed inductive study approach,^28^ we used Billett's concept of guidance^20^ and previous research into interprofessional guidance^22^, ^23^ to shape our research questions, theoretical framework, interview guide and analysis. Second, using a theory‐informing inductive data analysis approach, we held several theories and bodies of literature (specifically, IPC and IPE research) as possible interpretive tools that could inform the study. For example, we considered applying principles of situated learning theory like learning as a product of participation and interaction to inform analysis.^29^, ^30^

First, following the steps of Reflexive Thematic Analysis,^27^ RES and WSdG familiarized themselves with the Dutch data. Next, RES and WSdG generated initial codes in English through inductively analysing three transcripts (one trainee, one attending physician, one nurse) independently and discussed their preliminary codes and approach to coding with the aim to reach consensus on the alignment of their coding. Thereafter, RES and WSdG each coded 6 transcripts and had regular meetings to discuss the evolving codes, sub‐themes and themes. As themes were constructed, they were reviewed with the entire research team. This required translating themes and sample data excerpts into English for review so analysis discussions could take place with non‐Dutch speaking team members. After reaching a stable code‐tree (see Appendix 2), WSdG finished coding the remaining transcripts (N = 15). Regular full team meetings were held to discuss data‐analysis and preliminary results. The results of this study were further constructed by comparing the data of the different respondent groups to get a holistic view on the phenomenon of guiding trainees to collaborate interprofessionally during workplace learning. Data‐analysis was supported by ATLAS‐ti (version 24.0.1).

All data was stored on a secured network‐based hard disk especially installed and formatted for safely storing research data. This data was only accessible to RES and WSdG.

### 2.6 Reflexivity

The research team consisted of three social scientists and one physician with expertise in medical education. More specifically, the team consists of an educational scientist with expertise in workplace learning and the interprofessional learning and teaching dynamic (RES), a retired educational scientist with expertise in faculty development in health professions education and extensive experience as an interviewer (WSdG), a pulmonologist with responsibility for postgraduate training in a non‐academic teaching hospital (FWJMS) and a social science researcher with expertise in interprofessional healthcare team collaborations (LV). RES and LV have previously collaborated on a conceptual work regarding interprofessional dynamics during workplace learning,^12^, ^31^ work which informed the design of this study. RES and WDdG have previous experience working on the original pilot study which informed the design of the current study and also data analysis. RES, WSdG and FWJMS have previously collected data within the current research site and so were familiar with some of the main educational structures and key informants in this setting. LV's analysis work on the study was shaped by her position as an experienced researcher in IPC, a scholar with broad expertise in social science theories, and an outsider to the Dutch clinical education context. To monitor the confirmability of our data and results, she drew comparisons between our data and (a) existing literature, and (b) various theories (e.g. landscape of practice). RES, WSdG and LV are experienced qualitative researchers. Through our shared and complementary expertise, we explored our research questions from conceptual, theoretical, methodological and clinical experience points of view.

### 2.7 Ethics

Ethics approval was granted by Dutch Medical Education Research Ethics Committee (file #2022.7.7).

## 3 RESULTS

All our participants shared examples of guiding trainees' learning about IPC in clinical practice being provided, both *intra*professionally (i.e. within profession guidance, in this case (senior) physician to (junior) physician) and *inter*professionally (i.e. between profession guidance, in this case nurses and APCs guiding medical trainees). However, the majority of guidance—be it *intra*‐ or *inter*professionally provided—was described as limited, implicit and informal. Participants explained that nurses and APCs provided more guidance on IPC than attendings. The limited *intra*professional guidance that was provided was mainly offered via implicit role modelling and formal prompts from the postgraduate medical education assessment system. *Intra*professional guidance was obstructed when an attending did not feel responsible for providing such guidance, and when IPC processes were vaguely defined or understood. On the other hand, *inter*professional guidance on IPC was talked about by participants as being task‐oriented and focused on topics ranging from role boundaries to working agreements. Nurses' guidance of trainees could be hampered by nurses' experiences of personal and hierarchical barriers impeding their ability to provide feedback to medical trainees. In the following, we further explore the characteristics of *intra‐* and *inter*professional guidance providing examples and quotes from our participants.

### 3.1 Intraprofessional guidance

Our study participants explained that intraprofessional guidance on IPC rarely occurred. When attendings were asked why they sporadically provided explicit guidance on IPC to medical trainees, they described IPC as *‘a natural process’* (A2), and *‘something that usually goes well and doesn't require attention’* (A2). This opinion was shared by attending physicians across seniority levels. When asked what the main reason was for not providing much guidance on IPC, attending physicians referred to focusing their guidance on the medical expert competence and seeing this as their *‘core business’* (A8).

If attendings provided deliberate IPC‐related guidance, it was the result of being prompted either by the institution's formal postgraduate assessment system or by learning (via direct observation or feedback) from nurses that a trainee was struggling with IPC. As this participant explained, such prompts were needed:

*Because as long as things are going well, you see that it is not being mentioned. But if there, if it chafes, if there is friction, then you enter into a conversation with [the trainee in question] (…) But as long as things are going well, I don't think the collaboration is addressed. (…) You often see that when there is friction, then you [the supervisor] do engage*. (A9)

All attending physicians implicitly role‐modelled effective IPC skills, as illustrated by this participant's comment: *‘My conviction is that if you just model it well, that people will follow* [your lead]’ (A8). When asked for examples, attendings described modelling IPC communication strategies and affirming physician's role boundaries to other members of the healthcare team.

While attendings self‐reported acting as role models, trainees explained that their attendings were predominantly absent from the clinical context (i.e. not physically present in the same clinical environment throughout the day). Consequently, trainees rarely witnessed attendings' IPC skills, and the IPC‐related feedback from attendings was described as largely unhelpful. Trainees reported that the feedback received about IPC consisted of short vagueries (e.g. *‘good collaboration, well collaborated, nice collaborating’* [T7]) or statements that conflated IPC with feedback on the communicator competence (T4). Furthermore, trainees focused on the formal feedback that would be included in their portfolio which was predominantly written by attendings; consequently, trainees tended to dismiss nurses' informal feedback since it carried little formal weight.

Several nurse participants articulated that it was the attending physician's responsibility to provide IPC‐focused guidance because, as this participant explained, *‘the supervisor is ultimately responsible for training the trainee’* (N4). On the other hand, some attendings and trainees explained that providing IPC guidance was not their responsibility but rather that of the designated residency programme director or other direct physician supervisors. As one participant stated, “[the residency program director] *is more concerned with doing our* [trainees'] *training. And the other staff members are concerned with the collaboration within the group of trainees and with attendings from other disciplines* (T6). Several attending physician participants felt that their educational efforts should be focused on guiding learners' development of the medical expert role.

### 3.2 Interprofessional guidance

All participants described nurses' guidance of trainees' IPC as being informal and task‐oriented, offered in response to day‐to‐day practice or to problematic situations. As this nurse explained, IPC guidance is not consciously highlighted; instead, it is part of nursing practices:

*I don't do that very consciously, I just do that automatically, it's in me. I don't think: oh, shit, I have to give feedback now. No, I think: something is going wrong with the patient, with the doctor, and with my communication, I'm going to let them know*. (N2)

This informal, task‐oriented approach was confirmed by trainees. This trainee participant noted that IPC guidance was embedded in the flow of patient care, but not formally:

*‘I do think there is always frequent feedback back and forth with nurses, for example, but that is not so concrete like we sit down and discuss ‘how do you think I am doing?’ But more* [it is] *like: “I had asked you this and that didn't happen.” Not necessarily very concrete feedback but of course it's about how you work all day. If they are not satisfied with that, you will hear about it’* (T11)

Although trainees and supervisors felt that the guiding role of nurses was more pronounced for junior trainees trying to learn the ropes, nurses reflected on how the rotational system of residency training created a continuous cycle of providing guidance to trainees at all levels of seniority:

[There is] *a lot of turnover in residents. Every period, you notice that a whole new batch arrives. Then you just notice again: all the agreements that were made then lapse or have to be taught all over again*. (N1)

While the majority of IPC guidance from nurses was offered directly, Nurses described occasionally going to the attending with feedback for or a complaint about a trainee. Only one attending physician described seeking out nurses' feedback about trainees' IPC skills. Interestingly, this attending held a formal educator role on the medical team that was focused on medical students and, as this excerpt illustrates, had a practice of surveying the atmosphere in the clinical environment:

*We* [A8 and another educator colleague] *walk around so much everywhere… Yes, we make three visits a day, but also in the evening we walk around again, or we sit with the nurses to ask if everything is going well. You notice. It's just hard to explain… You just notice it, because this is not the atmosphere that's normally there. And then you start asking, and so it turns out there is indeed something going on in the communicative or collaborative aspect of that particular trainee*. (A8)

When nurse and APC participants were asked about their IPC‐focused guidance of medical trainees, they offered detailed descriptions of the range of topics they addressed in this educational work. First, nurses explained that they offered IPC guidance about the team and the clinical context, specifically by addressing professional roles and role boundaries, department processes, their preferred ways of working, working agreements and the unique nurses' perspective.

*“So then it's more guiding actually. To make the residents aware that it's not just the patient but that there are many more parties involved. And that it's important to coordinate care between them.”* (NP1)

As one nurse's story illustrates, the way in which that guidance is received by the trainee is variable:

*Yes, well the other day I had another example, there was a doctor who indeed had not come to do a round at 3 PM so I had already called him a couple of times, I had called him again and I said: “Are you coming to the ward for the 3 PM round?”* [the Trainee's reply was:] *“Yes, but we can also phone.” So I said: “Well, I'd rather you came to the ward then,” and that's when we finally gave it back to the* [trainee in question]: *“Our agreement here is that you come back to the ward at three o'clock, that you just make the round, determine the patient care plan again,” and yes, that's how you actually give it back to the person* [trainee] *in a normal way and yes, sometimes it is well received, sometimes it is less well received*. (N3)

In contrast to the attitude expressed by most attending participants, nurses felt that guiding trainees on IPC skills was, at least in part, their responsibility. However, the nurses were keenly aware that the power of their guidance was limited, that they did not have *‘as much influence as attending physicians’* (N2). As another nurse participant explained, the guidance of medical trainees by nurses was not formally recognized by the hospital since it was not reflected in nurses' formal duties.

*Our circle of influence as nurses in* [medical] *trainees' training is, of course, a bit smaller than that of a supervisor* [attending physician]. (…) *I definitely think we have influence, but I don't see it reflected in my duties*. (N4)

Nurses did not universally experience their IPC‐focused guidance of medical trainees as easy. In fact, several examples were provided by nurses of having to learn how to overcome professional hierarchical barriers. Nurses described coaching each other about how to give feedback to medical trainees and how to respond to situations where those trainees were non‐responsive to their feedback. However, as this data excerpt illustrates, nurse participants acknowledged that nurses were increasingly willing to speak up:

*I do think that* [compared to when I just started] *we as nurses do speak up more now and are also more willing to raise the alarm like, “gosh, yes, you know, I'm not going to spend half an afternoon waiting for you to come and tell me what needs to be done”, so to speak. So I do think that the nurses have stood up for themselves more in that, and that in the past maybe that hierarchy, that that played more of a role*. (7)

## 4 DISCUSSION

Helping medical trainees to be interprofessionally collaboration‐ready is an important goal of both undergraduate and postgraduate medical education.^1^, ^3^ Although undergraduate IPE has been harnessed as the foremost approach to attaining this goal, the transfer of what is learned during undergraduate IPE to clinical practice has been limited.^5^, ^6^, ^7^ Informed by research suggesting that learning to collaborate interprofessionally may be more successful during workplace learning^5^, ^32^, ^33^ but requires the guidance of more experienced members of the healthcare team, this study set out to better understand 1) to what extent guidance was provided to medical trainees in learning to collaborate interprofessionally during workplace learning and 2) who provided this guidance and how. Our results suggest that both intraprofessional and interprofessional guidance are taking place albeit infrequently, informally and implicitly. This finding echoes existing research aimed at optimizing interprofessional learning in the workplace.^34^ Our participants reported that nurses and APCs felt a stronger need to provide guidance to medical trainees than attending physicians. However, nurses and APCs faced barriers in shaping this role. Moreover, trainees were predominately focused on feedback coming from attending physicians and not as sensitive to feedback originating from nurses or APCs. When we frame these findings in relation to existing research and theory, important implications of our results are evident.

### 4.1 Role modelling to support interprofessional collaboration

Although research suggests that learning to collaborate interprofessionally through workplace learning has great potential,^4^, ^32^, ^33^ there is ample evidence that demonstrates how interprofessional workplace learning still fails to meet its promise.^5^ The results in our study echo these findings and suggest that this failure may be due to a lack of deliberate and explicit guidance on learning to collaborate interprofessionally. Workplace learning requires supervising, teaching and guiding trainees to navigate learning through everyday clinical practice^18^, ^19^; however, attending physicians in our study reported that learning to collaborate interprofessionally did not have to be a focal point of their supervision, teaching, or guidance. IPC was described as naturally occurring, only needing attention when it went awry. Some participants even rejected the notion that addressing this competency fell under their prevue; instead, they positioned that responsibility squarely on the shoulders of the residency programme director. We must consider the message such perceptions send to medical trainees. If implicit role modelling by attending physicians sends the message that IPC is not important, trainees will internalize these values.^35^ Therefore, reshaping the perceptions of and educational responsibilities for IPC is needed if we are to reap the benefits of interprofessional collaboration. As several authors have suggested, it is important to include IPC in our story‐telling and verbalisation^13^, ^36^ and in role modelling that demonstrates collaborative intentionality.^37^ Only then will our expectations and actions mirror what is currently only the *abstract* importance of IPC.

### 4.2 Opening eyes to the value of interprofessional feedback

Results of our research also suggest that the medical trainees in our study are more focused on becoming members of the community of physician practice (i.e. meeting or exceeding the expectations of the attending physicians) as opposed to becoming members of the community of healthcare practice.^12^ This was evidenced by trainees' strong focus on the feedback they received from attendings through their online portfolio which often lacked specific feedback on their IPC competencies. This focus on the community of physician practice echoes results from Jansen et al which noted how everything residents did during workplace learning was, besides being focused on patient care, in service of demonstrating their competence to attending supervisors.^38^ Similarly, the medical trainees in our study demonstrated being less open to the ‘cues and clues’^13^ ^, p.127^ of nurses and APCs during collaborative work in the healthcare team. In some instances, the medical trainees did not even recognize feedback when offered by nurses or APCs. Medical trainees' undervaluing of feedback from non‐physician healthcare team members is a well‐known phenomenon.^11^, ^39^ However, our study suggests that the effects of this phenomenon may be exacerbated by the online portfolio system. The extent to which interprofessional feedback is gathered within programmatic assessment and competency‐based medical education is often limited to multisource feedback forms^40^ or specific entrustable professional activities.^41^ This risks sending medical trainees the message that the perspectives of other healthcare professionals do not matter as much as the perspectives of physicians—even despite the fact that the trainees spend more time with the nurses and APCs than with attending physicians. Ideally, this incongruence should spur medical educators to consider re‐examining and re‐building evaluation practices to give more formal recognition to the feedback offered to medical trainees by nurses and APCs. At a minimum, medical trainees should be stimulated to critically reflect^42^ on IPC and their role in the landscape of healthcare practice^12^, ^43^ both during workplace learning as well as through the formal, postgraduate curriculum and assessment system.

### 4.3 Stimulating awareness and empowerment of nurses and APCs

Nurses and APCs were clearly an important source of guidance on IPC for medical trainees, but these clinicians often lacked awareness of their role. A recent study suggested that this lack of awareness was related to the extent to which nurses felt it was (or wasn't) their responsibility to provide guidance to medical trainees.^22^ Nurses who felt this responsibility saw medical trainees as future colleagues, whereas nurses who did not feel this responsibility referred to the work of guiding medical trainees as being outside of their job description and as supplementary work that they were too busy to voluntarily fulfil.^22^ Clearly, current pressures on healthcare systems worldwide do not invite substantial extension of work responsibilities for any member of the healthcare team. However, besides the level of awareness held by nurses, the perceived threshold to provide feedback due to professional hierarchical barriers between medical trainees and nurses also factored into how and if nurses provided guidance on IPC. Varpio and colleagues in their study on the contributions of nurses to residents informal workplace learning already highlight how nurses clearly felt the power differential between themselves and residents, even though residents may be less experienced within a given clinical setting.^21^ To capitalize on the role of nurses and APCs in guiding medical trainees in learning to collaborate interprofessionally, these clinicians need to be empowered. Despite recent initiatives within the context of our participants to explicitly communicate the importance of IPC, the understanding of and skills to address this in practice were still lacking. Research suggests that attending physicians have a key role in offering empowerment to nurses in their role as guides of medical trainee learning^11^, ^22^ since they act both as gatekeepers and knowledge brokers when it comes to nurses' role in medical trainees learning. Thus, to take full advantage of the feedback to medical trainees that nurses and APCs can offer (i.e. insights from people who have the most “boots on the ground” time with medical trainees in clinical learning environments)^44^ requires that attending physicians deliberately invite the interprofessional team in the feedback process and provide them with legitimacy,^22^, ^37^, ^43^, ^45^ and that formal procedures through which the interprofessional team is invited to provide feedback are redesigned to value those contributions.^46^ Furthermore, within the field of nursing, ‘rebel nurse leadership’ has been identified as a practice by nurses to challenge suboptimal practices and the status quo.^47^ This research suggests that nurses should more actively bring concerns forward to nurse managers to enable more durable change,^47^ something which could also be relevant to nurses' guidance medical trainees on IPC competencies.

### 4.4 Strengths and limitations

This study has strengths and limitations. A clear strength is the involvement of nurses, APCs, trainees and attending physicians; through their participation, we were able to compare and contrast their experiences with intra‐ and interprofessional guidance of medical trainees in learning to collaborate interprofessionally. By providing a detailed description of the context in which this graduate training programme was situated, we hope to have heightened the transferability of this work. This study was limited by having only nurses and APCs represent the voices of the broader interprofessional healthcare team. Future research should explore the breadth of the entire interprofessional healthcare team to gain a deeper understanding about interprofessional guidance during workplace learning. Although using the lens of guidance as described by Billett can be considered as a strength, future research should explore alternative theoretical lenses (e.g. Boundary Crossing^48^ and Cultural Historical Activity Theory^49^) to enrich our understanding.

## 5 CONCLUSIONS

This study contributes to a large body of research which suggests that learning to collaborate interprofessionally through workplace learning is high in potential but currently fails to meet its promise. We contend that its potential could be strengthened by more deliberate attention to both intra‐ and interprofessional guidance of medical trainees. By stimulating more deliberate physician role modelling, creating avenues for acceptance of interprofessional feedback and fostering awareness and empowerment of the interprofessional healthcare team, the potential of workplace learning towards IPC competence development may meet its promise.

## AUTHOR CONTRIBUTIONS

RES made substantial contributions to the conception and design, acquisition and interpretation of data and drafting the article. WSdG and FWJMS made substantial contributions in the acquisition and interpretation of data and drafting of the article. LV made substantial contributions to the conception and design, interpretation of data and drafting the article. All authors give their final approval of the version to be published.

## CONFLICT OF INTEREST STATEMENT

The authors declare no conflicts of interest.

## ETHICS STATEMENT

Ethical Approval was granted by the Dutch Association for Medical Education Ethical Review Board (file #2022.7.7).

## Supporting information


**Appendix 1.** Example interview guide.
**Appendix 2.** Code – Tree

## Data Availability

The data that support the findings of this study are available on request from the corresponding author. The data are not publicly available due to privacy or ethical restrictions.
