# Supplementary material for: Guiding medical trainees' workplace learning for interprofessional collaboration—Looking to physicians or seeing nurses?
Source: Med Educ. 2025 Feb 8;59(9):950–9. doi: 10.1111/medu.15617 (PMC12355634; doi:10.1111/medu.15617)
Supplement: Supplementary file 1 — Appendix 1. Example interview guide. Appendix 2. Code – Tree [file MEDU-59-950-s001.docx]

**Appendix 1 – Example interview guide**

*All participants*

To get a better idea of the processes in your department:

- Within which processes in your department does interprofessional collaboration play an important role?
- What do you understand interprofessional collaboration to be? What formal and informal dimensions are involved? What do you find particularly important in this?

*Attending Physicians | Nurses | APCs*

In your opinion, who is actively involved in teaching medical trainees to collaborate interprofessionally? In what way are these people involved?

- To what extent is this (getting involved in training/learning to work together) a formal process and/or an informal process?
- Could you give an example of times when there is interprofessional supervision in this area?

*Medical trainees*

Who is actively engaged in teaching you to collaborate in an interprofessional care team?

- In what ways do they do this?
- What is the role of different members of the healthcare team in this?

*All Participants*

Listing the different CANMEDS (medical expert, communicator, collaborator, health promoter, researcher, organiser, professionalism and quality) which of these roles do you pay most attention to in your supervision? *(Medical trainees: is most attention paid to during your training)* Why?

How does this attention relate to the attention you pay to the role of collaborator?

- When do you pay attention to the role of collaborator?
- In what ways do you pay attention to the role of collaborator?
- Do you think it is important to actively pay attention to the role of collaborator? Why?

*Attending Physicians | Nurses | APCs*

When it comes to learning to collaborate within the healthcare team and with other healthcare professionals, what, would you say, is particularly important to learn for more junior versus older trainees? Why?

- What do they need to know?
- What should they be able to do?
- What makes you notice when a medical trainee gets better at interprofessional collaboration?

*Medical trainees*

When it comes to learning to collaborate within the care team and other professions, what, would you say, is particularly important to learn? Why?

What is important to know?

What is important to be able to do?

To what extent do you pay attention to interprofessional collaboration in your supervision of medical students/trainees less experienced than you? How?

**Appendix 2: Code – Tree**

**Intraprofessional Guidance**

- Not deliberate
  - Implicit
  - At a distance
- Trainees more attuned to intraprofessional guidance than interprofessional guidance
- Prompted by PGME assessment system
  - Limited feedback on feedback forms
- Prompted when something is not going well
  - Prompted by information/feedback of nurses/APCs
  - Observing others working around the trainee
- Role modelling
  - Implicit
  - Communication strategies
- Physician’s role boundaries
- Responsibility to guide IPC
  - Not my responsibility
  - Responsibility of the residency program director
- Medical trainees creating IPC learning opportunities for junior trainees

**Interprofessional Guidance**

- Nurses & APC’s guidance on IPC
  - Informal
  - Task-oriented
  - Shaped by day-to-day practice
  - When something is not going well
- Feedback
  - Direct and formal feedback
    - Multi-source feedback round
    - Sporadic
  - Indirect and Informal feedback
    - Sought out by attending physician
    - Going to the attending physician to give feedback about a trainee
  - Feedback focus
    - Role boundaries
    - Inducting trainees into department
    - Preferred ways of working
    - Working agreements
    - Nurses’ unique perspective on patient care
  - Trainees’ feedback reception
    - Feedback not noticed
    - Poorly received
  - Giving feedback not easy
    - Hierarchical barriers
    - Nurses coaching nurses to give feedback
    - Increase in willingness to speak up
- Nurses/APCs awareness of guiding role
  - Interview prompted awareness
  - Feeling responsibility to train future colleagues
